# Natural History of Meningioma Development in Mice Reveals: A Synergy of *Nf2* and *p16*^Ink4a^ Mutations

**DOI:** 10.1111/j.1750-3639.2007.00105.x

**Published:** 2008-01

**Authors:** Michel Kalamarides, Anat O Stemmer-Rachamimov, Masaya Takahashi, Zhi-Yan Han, Fabrice Chareyre, Michiko Niwa-Kawakita, Peter M Black, Rona S Carroll, Marco Giovannini

**Affiliations:** 1 Inserm U674Paris, France; 2 Université Paris 7—Denis Diderot, Institut Universitaire d'hématologieParis, France; 3 AP-HP, Hôpital Beaujon, Service de NeurochirurgieClichy, France; 4 Molecular Neuro-Oncology and Pathology Department, Massachusetts General Hospital and Harvard Medical SchoolBoston, Mass; 5 Radiology, Beth Israel Deaconess Medical Center, Boston and Harvard Medical School,Boston, Mass; 6 Department of Neurosurgery, Brigham & Women Hospital, Harvard Medical SchoolBoston, Mass

## Abstract

Meningiomas account for approximately 30% of all primary central nervous system tumors and are found in half of neurofibromatosis type 2 patients often causing significant morbidity. Although most meningiomas are benign, 10% are classified as atypical or anaplastic, displaying aggressive clinical behavior. Biallelic inactivation of the neurofibromatosis 2 (*NF2*) tumor suppressor is associated with meningioma formation in all NF2 patients and 60% of sporadic meningiomas. Deletion of the *p16*^INK4a^/*p14*^ARF^ locus is found in both benign and malignant meningiomas, while mutation of the *p53* tumor suppressor gene is uncommon. Previously, we inactivated *Nf2* in homozygous conditional knockout mice by adenoviral Cre delivery and showed that *Nf2* loss in arachnoid cells is rate-limiting for meningioma formation. Here, we report that additional nullizygosity for *p16*^Ink4a^ increases the frequency of meningioma and meningothelial proliferation in these mice without modifying the tumor grade. In addition, by using magnetic resonance imaging (MRI) to screen a large cohort of mutant mice, we were able to detect meningothelial proliferation and meningioma development opening the way to future studies in which therapeutic interventions can be tested as preclinical assessment of their potential clinical application.

## INTRODUCTION

Meningiomas account for approximately one-fourth of all primary central nervous system tumors. They usually affect older adults, particularly women, and are often associated with significant morbidity (13). Although most meningiomas are benign, World Health Organization (WHO) grade I lesions, 10% of meningiomas are classified as atypical (WHO grade II) or anaplastic (WHO grade III) (10), displaying aggressive clinical behavior and leading to increased patient morbidity and mortality (27). One of the genetic mechanisms underlying meningioma tumorigenesis includes early inactivation of the neurofibromatosis 2 (*NF2*) tumor suppressor gene (12, 18). Previously, we inactivated *Nf2* in homozygous conditional knockout mice by adenoviral Cre (*adCre*) delivery into arachnoid cells through the cerebrospinal fluid (CSF) of newborn mice (6). We have shown that biallelic loss of *Nf2* is rate-limiting for meningioma development with mice developing a range of meningioma subtypes histologically similar to WHO grade I human meningiomas.

In humans, higher-grade meningiomas are aggressive and are associated with poor clinical outcome and with frequent recurrence after surgery and radiotherapy. So far, only few of the individual genes affected by the chromosomal aberrations found in atypical and malignant meningiomas are known. Deletion of the *p16*^INK4a^/*p14*^ARF^ locus or monosomy of chromosome 9 was found in 17% of grade I, 52% of grade II, and 74% of grade III meningiomas (15) resulting in loss of p16 expression (23). The majority of grade III meningiomas either show homozygous deletions of *p16*^INK4a^,*p14*^ARF^, and *CDKN2B*, mutations in *p16*^INK4a^ and *p14*^ARF^, or lack of expression of one or more of these genes (3). The *p16*^INK4a^/*p14*^ARF^ locus encodes two distinct cell cycle inhibitory proteins, *p16*^INK4a^ and *p14*^ARF^ (*p19*^Arf^ in the mouse) by alternative first-exon usage and alternative reading frames (20). *p16*^INK4a^ arrests cells in the G1 phase of the cell cycle by binding the cyclin-dependant kinases CDK4 and CDK6 and inhibiting their ability to phosphorylate and inactivate the retinoblastoma (RB1) family of tumor suppressor proteins (22). *p14*^ARF^ acts through MDM2 to stabilize and activate the key checkpoint protein p53, which can arrest cells in both G1 and G2 or induce apoptosis (20). In the present study, we evaluated the effects in meningioma initiation and progression of *p16*^Ink4a^ loss with retention of *p19*^Arf^ in synergy with *Nf2* loss.

In addition to dissecting the molecular changes associated with tumor initiation and progression, genetically modified (GEM) cancer models can be used as “filters” for the screening and selection of therapeutic agents for human trials (5), and are particularly valuable for rare tumors types, such as brain tumors (2). However, evaluating therapeutic agents in a pertinent mouse model of meningioma is challenging because of low tumor penetrance and to their relatively slow growth rate. Thus, small animal imaging is required to monitor tumor growth and response to therapeutic modalities. As in humans meningiomas are routinely detected and followed by magnetic resonance imaging (MRI), we sought to determine whether small animal MRI could detect abnormal meningeal proliferations in the living mouse. As an initial step toward using these mice as a preclinical model of meningioma, we have defined an imaging protocol based on gadolinium enhancement to delineate tissue morphology and pathology, as used for human meningiomas.

## METHODS

### Mice

To obtain *Nf2*^flox2/flox2^*;p16*^Ink4a*/*^ mice, *Nf2*^flox2/flox2^ mice (4) on the FVB/N background were bred to *p16*^Ink4a*/*^ mice (11) on a FVB/N/129Ola mixed background, and the resulting *Nf2*^flox2/+^; *p16*^Ink4a*/+^ mice were intercrossed. All mice used for this analysis were predominantly FVB/N mixed strain with contemporaneous littermates serving as controls. Pathogens were tested on a quarterly basis, and all serologies tested were negative throughout the study. All animal care and experimentation reported herein were conducted in compliance with the guidelines and with the specific approval of Institutional Animal Care and Use Committee of the French Department of Agriculture.

### Injection of adenoviruses

Cre recombinase was targeted to the mouse leptomeninges by direct injection of 3 µL (1 × 10^8^ plaque-forming units) of *adCre* suspension into the subarachnoidal space on postnatal day 2 by trans-orbital or subdural approach, as previously described (6).

### Histopathology and immunohistochemistry

Mice were sacrificed by CO_2_ inhalation when seriously ill (rarely) or after the last magnetic resonance (MR) image acquisition at 15 months and a necropsy was performed. Histopathological analysis was performed as previously described (6).

PGDS immunohistochemistry was carried out on adjacent paraffin sections using affinity-purified rabbit polyclonal antibodies (1:500; Santa Cruz, CA, USA sc-14825) and standard techniques (DakoCytomation, Glostrup, Denmark). The terminology used for the description of the meningothelial lesions in the mouse models relies on the WHO classification of human tumors as a reference (13). The term “meningothelial hyperplasia” is not used, as in humans that refers to the proliferation of reactive, normal arachnoid cells. “Meningothelial proliferation” refers to very small (microscopic) lesions composed of meningothelial cells that represent early tumor formation. “Meningioma” refers to a larger lesion, with features similar to a WHO grade I meningioma in human. “Meningioma *en plaque*” refers to a meningioma with a growth pattern of diffuse thickening of the dura, similar to meningioma *en plaque* in humans.

### Electron microscopy

Paraffin-embedded tissue was cut into nine pieces, placed in xylene overnight, rehydrated in graded ethanols (100% to 25%), and then placed in gluteraldehyde. Subsequently, the tissue was post-fixed in osmium tetroxide, stained with uranyl acetate, dehydrated in graded ethanol solutions, infiltrated with propylene oxide/Epon mixtures, flat embedded in pure Epon, and polymerized overnight at 60°C. One-micrometer sections were cut, stained with toluidine blue, and examined by light microscopy. The best section containing meningioma was chosen to proceed for electron microscopy study and trimmed accordingly. Thin sections were cut with an LKB8801 ultramicrotome and diamond knife, stained with Sato's lead, and examined in a Phillips 301 transmission electron microscope.

### MRI

MRI was used to identify and localize meningiomas. MR study was performed on a 4.7 Tesla microimaging system (Biospec, Bruker BioSpin MRI, Inc., Ertlingen, Germany). The system consists of three-axis self-shielded magnetic field gradients, with 30 G/cm maximum gradient amplitude in all three channels. Under anesthesia by inhalation of 1%–2% of isoflurane (IsoFlo®, Abbott Laboratories, Abbot Park, IL, USA), all animals were intraperitoneally injected (1) a MR contrast agent, Gd-DTPA (Magnevist®, Schering, Leverkusen, Germany) at a dose of 0.8 mL/kg body weight and placed 15 minutes later in the radio frequency coil (inside diameter 35 mm). First the localizer imaging with a first echo sequence (rapid acquisition with relaxation enhancement: RARE) was conducted on three orthogonal axes. Subsequently, transverse T2-weighted images (T2-WIs) and T1-weighted images (T1-WIs) were acquired with a RARE and a conventional spin-echo sequence, respectively, on entire brain. A pulse repetition time and echo time were 2000 and 40 ms for T2-WI and 500 and 11 ms for T1-WI. Other parameters were: 3 cm field of view, 128 × 128 matrix size, eight (for T2-WI) and four (for T1-WI) averages, resulting in a total scan time of approximately 2.4 and 4.5 minutes, respectively. Some relevant mice were reimaged before injection of contrast agent to get T1-weighted images without contrast enhancement. After the brain imaging, some animals that showed neurological symptoms were repositioned and subjected to spinal cord imaging. After the localization, spinal cords were imaged in 1 mm sagittal sections. The other imaging parameters were the same as those in the brain imaging described above. The MRI findings were qualitatively analyzed by two experienced investigators, M.K. and M.T., with consensus. MR studies were repeated at 6, 9, 12, and 15 months followed by pathological examination of all mice.

### Statistics

Significant differences in survival and tumor development were identified using χ^2^ test; *P* < 0.05 was considered significant.

## RESULTS

### Additional nullizygosity for *p16*^Ink4a^ increases the rate of meningioma development and meningothelial proliferation in *adCre;Nf2*^flox2*/*flox2^ mice

**Figure 1:**
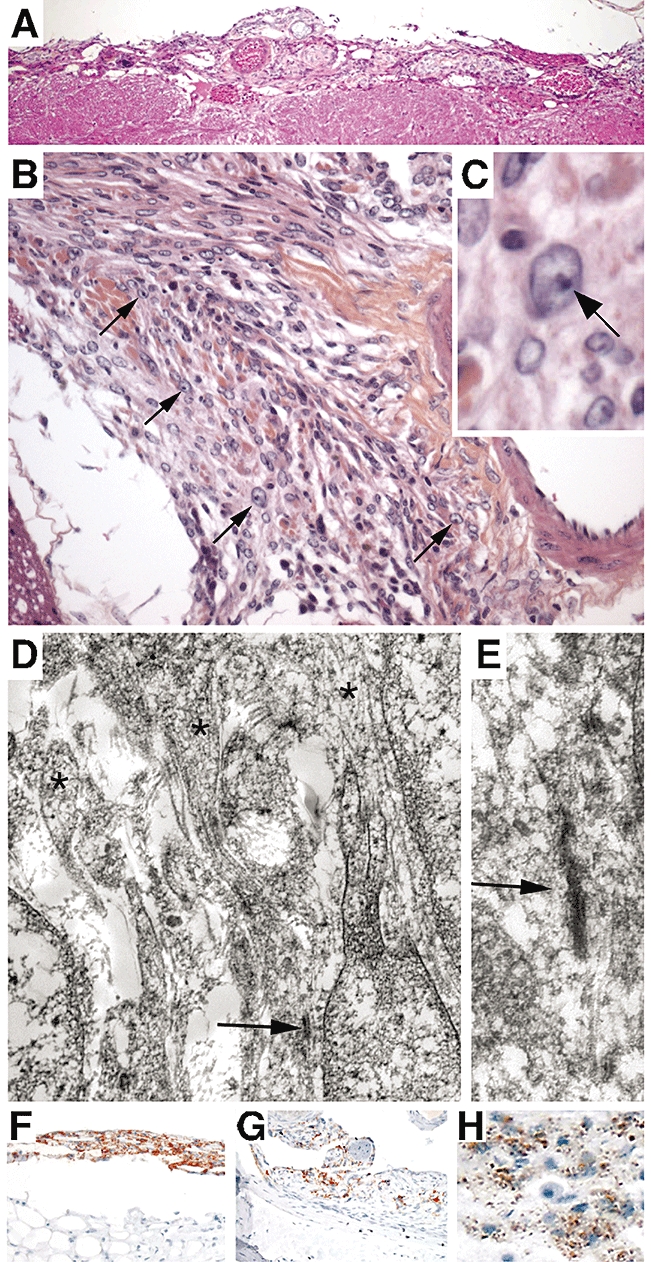
Pathological characterization of murine meningiomas A. Hematoxilin and eosin-stained section showing a transitional meningioma *en plaque* overlying the brainstem. B,C. Transitional meningioma covering the right trigeminal nerve. Some cells have prominent nucleoli, reminiscent of atypical features in human tumors (arrows). D. Ultrastructural study of a large transitional spinal meningioma, using paraffin-embedded material, demonstrated features characteristic of human meningioma. The arrow indicates tight junctions and asterisks show complex interdigitating cell processes. E. Detail (higher magnification) of the image in D. Immunostaining for Prostaglandin D2 synthase (PGDS) in murine meningiomas. Mouse meningiomas show two different PGDS staining patterns. F. Transitional meningioma showing multifocal process with both meningioma *en plaque* and discrete nodules. The “*en plaque*” part of the tumor shows multiple layers with strong cytoplasmic staining. G. Fibroblastic meningioma with regional positivity for PGDS. In some areas, the cytoplasmic staining is dispersed with few, abnormally distributed granules. H. Higher magnification reveals PGDS immunoreactive granules dispersed in the cytoplasm.

To investigate the potential synergy of *Nf2* inactivation and homozygous *p16*^Ink4a^ mutation in meningioma development, a cohort of 32 *Nf2*^flox2/flox2^;Ink4a*^/^* mice were injected with *adCre* and compared with 80 *adCre*-injected *Nf2*^flox2/flox2^. As control groups, 10 *adLacZ*-injected *Nf2*^flox2/flox2^ mice and eight *adCre*-injected FVB/N WT mice were used. The survival of *adCre;Nf2*^flox2/flox2^;Ink4a*^/^* mice was not reduced compared with that of *adCre;Nf2*^flox2/flox2^, but the rate of meningioma development was significantly higher in *adCre;Nf2*^flox2/flox2^;Ink4a*^/^* (10 of 27 histologically examined mice; 37%) compared with *adCre;Nf2*^flox2/flox2^ (9 of 72 histologically examined mice; 13%) mice (χ^2^, *P* < 0.01). The other phenotypic abnormalities are listed in Table 1. Histologically, all the meningiomas had features of benign neoplasms (WHO grade I) similar to benign human meningiomas of meningothelial, transitional, psammomatous, or fibroblastic histological subtypes. Two patterns of growth were identified in mouse meningiomas: a diffuse thickening meningioma *en plaque*-like pattern (Figure 1A), and a discrete nodular pattern (Figure 1B). In two of 10 meningiomas found in *adCre;Nf2*^flox2/flox2^;Ink4a*^/^* mice, features that in human tumors may be called “atypical” (one feature in each tumor) were observed: prominent nucleoli (Figure 1C) and crowding of the cells, but neither had enough atypical features to be called atypical meningioma by the current WHO classification of human tumors.

**Figure 2:**
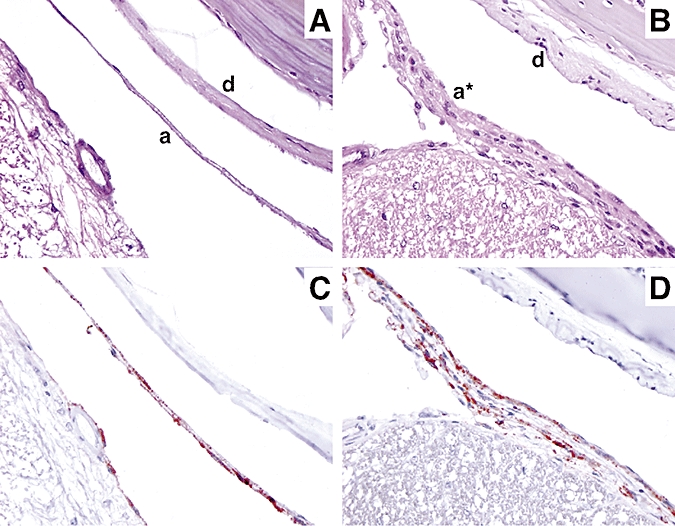
Pathological characterization of meningothelial proliferation in mice Hematoxilin and eosin-stained sections showing (**A**) a normal arachnoid monolayer (a) and dura mater (d) and (**B**) meningothelial proliferation (a*) characterized by multiple layers of arachnoid cells. (**C**) Immunostaining for Prostaglandin D2 synthase (PGDS) of mouse normal leptomeninges and (**D**) meningothelial proliferation. Similar PGDS staining patterns of normal arachnoid and meningothelial proliferation showing intracytoplasmic granules with perinuclear distribution.

Diagnostic ultrastructural features of meningioma, including interdigitating cell processes and desmosomal intercellular junctions, were found in one *adCre;Nf2*^flox2/flox2^^2^;Ink4a*/* mouse meningioma analyzed by electron microscopy (Figure 1D,E). Human arachnoid cells specifically express Prostaglandin D2 synthase (PGDS) and intense PGDS immunopositivity is observed in about 100% of human meningothelial and 50% of fibroblastic meningiomas (8, 29). We found the PGDS staining pattern of normal arachnoid in mice is similar to that observed and reported in humans (data not shown). The immunostaining for PGDS in normal arachnoid cells, in both human and mouse, shows intracytoplasmic granules in perinuclear distribution. Almost all mouse meningiomas showed some positive staining. In some cases strong staining was seen within some regions of the tumor (patchy distribution) (Figure 1F), and in other tumors, only few scattered positive or weakly positive cells were present in the tumor (Figure 1G). Interestingly, in most cases PGDS staining of tumor cells (Figure 1H) was different from that seen in the adjacent normal arachnoid cells (Figure 2A,C) and in meningothelial proliferation (Figure 2B,D): with fewer, fine granules dispersed in the cytoplasm the pattern of diffuse cytoplasmic staining (rarely perinuclear). In some cases, at the injection site there was a diffuse thickening of the arachnoid, which may represent either a diffuse meningioma *en plaque* or meningothelial proliferation (early tumor), an lesion that we previously associated with mouse leptomeningeal tumorigenesis (6). We excluded a reactive process from the differential diagnosis of these subdural thickenings as these patterns were never observed in the control cohorts of *adLacZ*-injected *Nf2*^flox2/flox2^ mice and *adCre*-injected FVB/N WT mice.

Meningothelial proliferation was observed more frequently in *adCre;Nf2*^flox2/flox^^2^;Ink4a*/* (21/27; 77%) than in *adCre;Nf2*^flox2/flox2^ (33/72; 46%) mice (χ^2^, *P* < 0.01). Consequently, hydrocephalus caused by meningothelial proliferation impeding CSF flow was more frequent in *adCre;Nf2*^flox2/flox^^2^;Ink4a*/* mice (Table 1).

**Table 1:** Summary of the phenotypic consequences of transorbital (t.o.) or subdural (s.d.) *adCre* injection in *Nf2*^flox2/flox2^ and *Nf2*^flox2/flox2^;Ink4 a*/* mice.

Phenotypic abnormality	*adCre;Nf2*^flox2/flox2^ (%)		*adCre;Nf2*^flox2/flox2^;Ink4a*/* (%)	
		
	t.o. (n = 40)	s.d. (n = 32)	t.o. (n = 13)	s.d. (n = 14)
Meningioma	6 (15)	3 (10)	5 (38)*	5 (36)*
Intracranial	6 (15)	3 (10)	5 (38)	2 (14)
Spinal	0	0	0	3 (21)
Meningothelial proliferation	19 (48)	14 (44)	11 (85)*	10 (71)*
Osteoma	22 (55)	19 (59)	9 (69)	12 (86)*
Osseous metaplasia (trigeminal nerve)	1 (2)	0	2 (15)	0
Liver tumor	3 (7)	2 (6)	2 (15)**	3 (21)**
Hydrocephalus	17 (43)	9 (28)	9 (69)**	6 (43)**

* χ^2^,*P* < 0.01;

** χ^2^, *P* < 0.05.

Other phenotypic abnormalities observed included osteomas at the *adCre* injection site, presumably induced by biallelic *Nf2* inactivation in a neural crest precursor (4, 6, 17), and liver tumors (6). The rate of osteomas and liver tumors (three hepatocellular and two cholangio-carcinomas) development was higher in *adCre;Nf2*^flox2/flox2^;Ink4a*/* mice (Table 1).

Altogether, these data indicate that in the mouse homozygous inactivation of *p16*^ink4^ tumor suppressor gene, with retained *p19*^Arf^ function, synergizes with *Nf2* loss in meningioma initiation, but not in meningioma progression.

### Development of a preclinical model for NF2-related meningiomas: murine meningiomas show radiological features of human meningiomas

**Figure 3:**
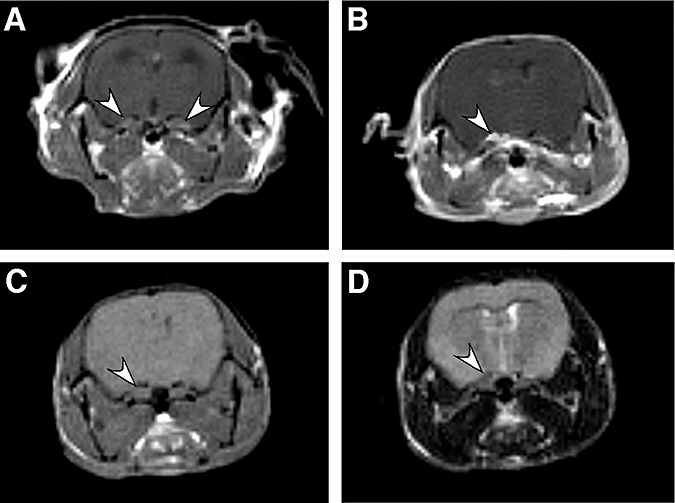
Murine intracranial meningiomas show common magnetic resonance imaging (MRI) features of human meningiomas. A right-side transorbital-injected *adCre;Nf2*^flox2/flox2^ mouse was followed by serial MRI **A.** Coronal T1-weighted image after Gd-DTPA injection at 12 months showing normal structures with moderate gadolinium enhancement of trigeminal nerves (arrowheads). **B.** Coronal T1-weighted image after Gd-DTPA injection at 15 months showing intense homogeneous Gd-DTPA enhancement above right trigeminal nerve (arrowhead) corresponding to a histologically confirmed right supratrigeminal transitional meningioma. (**C**) Coronal T1-weighted image and (**D**) T2-weighted image of the meningioma in **B** showing typical MRI features of human meningiomas: homogeneous isointensity with cerebral cortex on non-enhanced T1-weighted and T2-weighted sequences (arrowhead).

**Figure 4:**
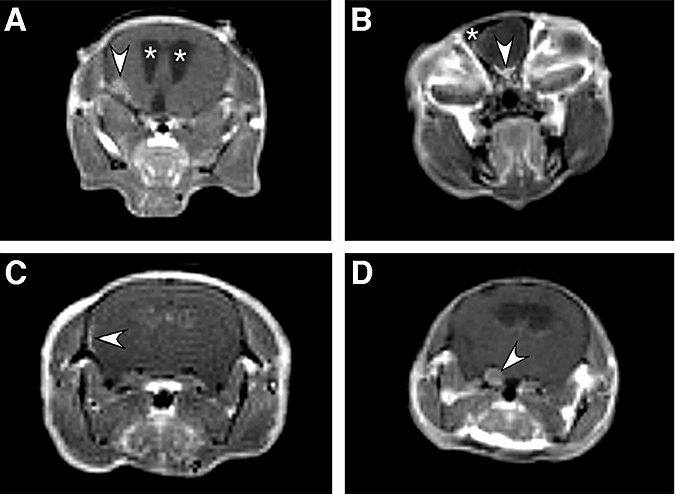
Some examples of coronal Gd-DTPA-enhanced T1-weighted magnetic resonance (MR) images after transorbital (**A,B,D**) or subdural (**C**) *adCre* injection **A.** Arrowhead indicates lateral round Gd-DTPA enhancement corresponding to a fibroblastic meningioma associated with hydrocephalus characterized by enlarged ventricles (asterisks). **B.** Arrowhead indicates abnormal, thick Gd-DTPA enhancement corresponding to a histologically confirmed meningothelial meningioma that would be defined as olfactory meningioma in human pathology. A hypointense signal corresponding to an osteoma is indicated by an asterisk. **C.** Meningioma *en plaque* nested within areas of meningothelial proliferation revealed by lateral linear thick Gd-DTPA enhancement (arrowhead). **D.** Imaging of a false-positive case of meningioma. Coronal T1-weighted image after Gd-DTPA injection showing moderate gadolinium enhancement of enlarged trigeminal nerves (arrowhead). Histological analysis revealed osseous metaplasia developing within the trigeminal nerve ipsilateral to the transorbital injection site.

Because meningiomas are routinely detected and followed by MRI in patients, we sought to determine whether small animal MRI could detect leptomeningeal pathology (meningothelial proliferations and meningiomas) in the living mouse. To correlate the histological findings with the radiological appearance of meningothelial proliferations and meningiomas, we analyzed 27 *adCre;Nf2*^flox2/flox2^;Ink4a*/*, 72 *adCre;Nf2*^flox2/flox2^ and 9 *adLacZ-*injected *Nf2*^flox2/flox2^ mice (as negative control) by serial MRI follow-up (6, 9, 12 and 15 months). At the end of the MRI follow-up period, all mice were sacrificed and processed for histological analysis. To determine if mouse meningiomas enhance with gadolinium-based magnetic resonance contrast agent (Gd-DTPA), as typically observed in human meningiomas, Gd-DTPA was injected intraperitoneally before MRI. Pathological gadolinium enhancement was observed in 75% of the intracranial mouse meningiomas (Figures 3 and 4). Isolated meningiomas (ie, without meningothelial proliferation, n = 8) showed strong gadolinium enhancement appearing as thick, linear, or rounded shapes (Figures 3B and 4A,B). These radiological features are similar to those of human meningiomas showing iso or hyposignal on T1-weighted sequences and strong and homogenous contrast enhancement (24). In some cases, surrounding edema, seen as hyperintensity on T2-weighted MRI, was observed. Some meningiomas *en plaque* were found nested within areas of meningothelial proliferation (n = 4), with images of linear gadolinium enhancement (Figure 4C). Meningothelial proliferation was identified in 70% of the cases by linear gadolinium enhancement and/or hydrocephalus. Hydrocephalus was always easily detected on T1- and on T2-weighted sequences (Figure 4A). Altogether, MRI allowed the diagnosis of meningothelial proliferation with a sensitivity of 70% and a specificity of 78% (Table 2).

**Table 2:** Correlation of pathological findings of meningioma and meningothelial proliferation with intracranial magnetic resonance imaging (MRI) appearance.

MRI result	Pathological finding		
	
	Meningioma n = 16	Meningothelial proliferation n = 54	No meningothelial proliferation n = 45
“Positive” gadolinium enhancement	8*	8†	3
Gadolinium enhancement and hydrocephalus	4	11	0
Hydrocephalus	0	19	7
Total	12	38	10
“Negative”	4	16	35

* Linear or rounded gadolinium enhancement.

† Linear gadolinium enhancement.

Radiological features of meningothelial proliferation (linear gadolinium enhancement and/or hydrocephalus) were present as early as 6 months and did not evolve over time. In contrast, an increasing incidence of meningiomas was observed at later time points: 9, 12, or 15 months (Figure 3A,B). Some histologically confirmed meningiomas were not identified by MRI (4/16; 25%). They corresponded to very small tumors in two cases and to completely calcified meningiomas in two other cases where, as expected based on experience in human pathology, no contrast enhancement was seen on T1-weighted sequences.

**Figure 5:**
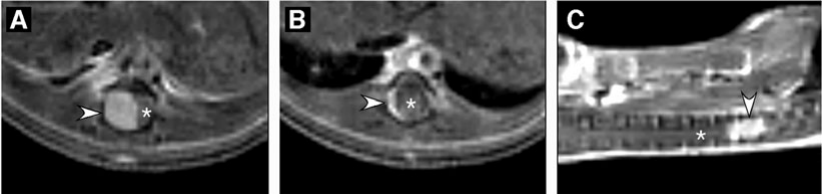
Murine intraspinal meningiomas show common magnetic resonance imaging (MRI) features of human spinal meningioma. This subdural-injected *adCre;Nf2*^flox2/flox2^;Ink4a*^/^* 8-month-old mouse rapidly developed paraparesis **A.** Axial T1-weighted image showing a large, histologically confirmed, Gd-DTPA-enhanced fibroblastic meningioma (arrowhead) compressing the spinal cord (asterisk). **B.** Remarkably, a tailing of the meningioma along the dura mater (arrowhead) was present at a lower level. A dural tail is often noted associated with human meningiomas and known as “dural tail sign”. **C.** Sagittal view of spinal cord (asterisk) showing the spinal meningioma (arrowhead).

In contrast, three false-positive cases corresponded to voluminous osseous metaplasia developing within the trigeminal ganglion ipsilateral to the transorbital injection site. Osseous metaplasia is presumably induced by biallelic *Nf2* inactivation in neural crest precursor cells as observed in conditional (P0) *Nf2* knockout mice (4). Thus, radiological criteria can be drawn allowing differential diagnosis of osseous metaplasia of the trigeminal ganglion vs. suprasellar meningioma: strong gadolinium enhancement of the enlarged trigeminal nerve vs. gadolinium enhancement overlying normal-sized trigeminal nerve (Figure 3D). As in our previous series, because of *adCre* diffusion through the CSF circulation, spinal meningiomas (n = 3) were found in *adCre;Nf2*^flox2/flox2^;Ink4a*/* mice and spinal MRI was performed in two mice presenting with paraparesis, a neurological signs suggestive of spinal cord compression (Figure 5A,C). Radiologically, these tumors, appearing as a dural-based mass with a dural tail (Figure 5B), presented the typical features of human spinal meningiomas compressing the spinal cord and enhancing brightly and homogenously with contrast.

## DISCUSSION

Deletion of the *INK4a-ARF* locus is one of the most frequent genomic alterations found in human high-grade meningiomas (15) encouraging the question of which protein provides the relevant tumor suppressor activity of the locus. Here we have shown that *Ink4a* nullizygosity enhances the incidence of meningioma formation induced by somatic *Nf2* loss in arachnoid cells, and that in mice *p16*^ink4a^ loss is not a critical event associated with malignant progression of meningioma.

The role of *Ink4a-Arf* in tumor suppression in mice has been extensively studied (21). Mice deficient for both *p16*^Ink4a^ and *p19*^Arf^ developed a variety of spontaneous tumors within their first year of life (19). This phenotype could to a large extent be attributed to the loss of *Arf* alone as the *Arf* null mice showed most of the same traits as the *Ink4a-Arf* null mice (7) and Ink4a*/* mice showed only a subtle predisposition to spontaneous tumor formation later in life (>15 months) (11). In this study we did not observe tumors related to *p16*^Ink4a^ loss as the end point was set at 15 months of age. It has been shown previously that *NF2* expression induces a decrease in cyclin D1 and CDK4 kinase activity, concomitant with dephosphorylation of pRB and reduced DNA synthesis (9, 28). Thus, the synergy we observed between *Nf2* and *p16*^Ink4^ mutations in meningioma development reflects the concomitant loss of two regulators of CDK4 activity resulting in cyclin D1 activation. Remarkably, meningiomas developed in double-mutant mice do not show features characteristic of high-grade meningioma suggesting that loss of the *p14*^Arf^ rather than the *p16*^Ink4a^ component of the locus is critical for malignant transformation of meningiomas. We have previously confirmed that in mice (6), as in humans (3) p53 inactivation does not synergize with *Nf2* loss in meningioma initiation and progression. Thus, the suppressive activity of *p14*^Arf^ on meningioma transformation is p53-independent. Overall, our data strongly support the view that one role of loss of *p16*^Ink4^ in meningiomagenesis could be to sensitize arachnoid cells to *Nf2* loss. Similarly, it has been suggested that one role of *Ink4a-Arf* loss in gliomagenesis could be to sensitize astrocytes to transformation through dedifferentiation in response to the appropriate oncogenic stimuli (26).

In order to facilitate the validation, comparative analysis with human tumors and use for drug screening in preclinical trials of the mouse models, we have used the WHO classification of human meningiomas as a reference (10). However, some important differences exist between the human and murine lesions. In humans the distinction of meningothelial hyperplasia, implying normal arachnoid cells that proliferate as a reactive process, from meningioma is difficult and relies mostly on clinicopathological features including absence of gross visibility, absence of dural invasion and of clinical symptoms, and history of local inciting factors such as trauma, hemorrhage, chronic inflammatory infiltrate, or adjacent tumor, or a systemic condition such as old age or chronic renal failure. Histologically meningothelial hyperplasia is defined by the presence of nests of 10 or more cell layers thick (16). In our mouse model although the lesions are small, they are not reactive, but neoplastic, as they are never observed in control mice at the site of injection. Therefore, in the mouse, we preferred the term early tumors or meningeal proliferations instead of meningeal hyperplasia.

Another difference that was noted was the high incidence of *en plaque* meningiomas in the mouse model. In humans, *en plaque* meningiomas represent an unusual growth pattern, manifested as a carpet-like smooth or nodular dural thickening, which does not raise above the *dura mater* but often invades adjacent skull, and is often associated with hyperostosis. *En plaque* meningiomas in humans are most common in the skull base, but may grow as diffuse, flat plaques over the convexities or form a circumferential mass around the spinal cord (14). The histological features of the mouse *en plaque* meningiomas were similar to those seen in humans, but were not associated with hyperostosis and had no site predilection. The higher frequency of *en plaque* meningiomas in our mouse model is likely caused by the multifocality of *Nf2* inactivation induced by diffusion in the subarachnoid space of the highly concentrated *adCre* vector suspension.

All the tumors in our mouse models were WHO grade I type lesions. Interestingly, two tumors in *adCre;Nf2*^flox2/flox2^;Ink4a*/* mice showed atypical features (prominent nucleoli, crowded cells). In humans these atypical features may imply a more aggressive clinical course. In the mouse, we have no clinical data to support or refute the significance of an atypical histological feature in a meningioma, but as this was a model in which synergic action of *Nf2* and *p16*^Ink4a^ inactivation was assumed, it is of interest to note the presence of histological features that may connote a more aggressive tumor. Analysis of a larger series of tumors would be needed to determine the relevance of this observation and may lead to the formulation of a modified WHO classification for mouse models, similar to the one formulated for peripheral nerve sheath tumors in mouse models (25).

Here we show also that, as in humans, MRI can be used to image meningiomas in the mouse. By using MRI to screen a large cohort of mice rather than a few “interesting” mice, we were able to determine the time-course of meningothelial proliferation and meningioma development. For screening, we imaged mice using a gradient echo sequence with short acquisition-time sequences (only 6 minutes). With this relatively short scan time, we could achieve sufficient signal–noise ratio and image spatial resolution to detect most meningiomas and meningothelial proliferations. To increase the sensitivity of meningioma detection by MRI, it would be necessary to increase image resolution, but a twofold increase of image resolution would result in an increase of scan time by four fold. Therefore, the balance between reasonable data acquisition time and adequate spatial resolution has to be considered. A crucial step in the translation of this mouse meningioma model into a preclinical model was the ability to detect meningothelial proliferation by MRI. Follow-up of these early meningiomas with a non-invasive imaging technique provides an additional readout in preclinical studies for chemoprevention of NF2-related tumors.

Finally, monitoring meningioma growth by MRI opens the way to future studies in which therapeutic intervention on sporadic meningioma can be tested in this mouse model as preclinical assessments of the potential for clinical application.
